# The complete mitochondrial genome of the banana pathogen *Fusarium oxysporum* f. sp. *cubense* M5

**DOI:** 10.1128/mra.00421-24

**Published:** 2024-09-09

**Authors:** Luis David Maldonado-Bonilla, Juan Caballero-Pérez, Rodolfo Enrique Ángeles-Argáiz

**Affiliations:** 1 Instituto de Genética, Universidad del Mar Campus, Puerto Escondido, Oaxaca, Mexico; 2 Bioinformatics Facility, Max Planck Institute of Immunobiology and Epigenetics, Freiburg im Breisgau, Germany; 3 Red de Manejo Biotecnológico de Recursos, Instituto de Ecología A.C., Xalapa, Veracruz, Mexico; University of California Riverside, Riverside, California, USA

**Keywords:** *Fusarium*, phytopathogens, mitochondria, intron, genome analysis, mitogenome

## Abstract

We report the complete mitochondrial genome of a causal agent of banana fusarium wilt isolated in Mexico. The whole set of genes encoding proteins related to respiration and ATP synthesis, rRNAs, tRNAs are enlisted. Two open reading frames of unknown function conserved in *Fusarium oxysporum* were also identified.

## ANNOUNCEMENT

*Fusarium oxysporum* f. sp. *cubense* is the causal agent of the fusarium wilt that hampers banana production worldwide (1). Fungal mitochondrial DNA (mtDNA) presents a high variation in the number and position of genes (2). However, the mtDNA has an overall low intra-species variation in the three lineages of *F. oxysporum*, although there are three variants of a large variable region (3) that can enable novel PCR-based strategies of diagnostics. *F. oxysporum* f. sp. *cubense* M5 (*Foc*M5) was isolated from diseased banana plants in southern Mexico (4).

**Fig 1:**
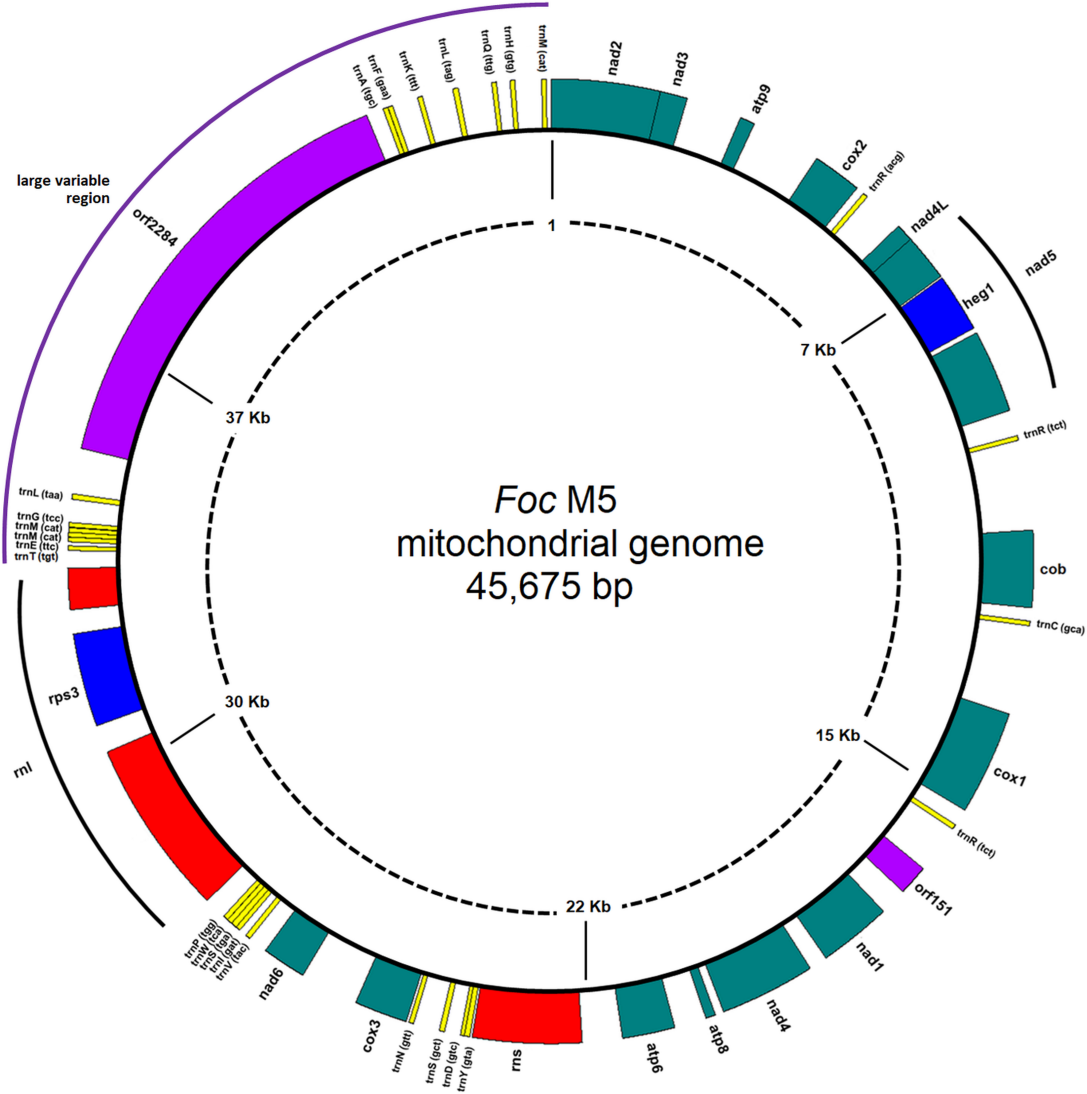
Mitochondrial genome of *Foc*M5. The diagram was generated by GenomeVx 2.0 (10). All the genes are oriented clockwise.

Mycelium from *Foc*M5 that grew for 2 weeks in potato dextrose agar plates covered with a cellophane disc at 25°C was harvested and grounded in liquid nitrogen. Later, the genomic DNA was extracted using the DNeasy Plant Mini Kit (QIAGEN). The DNA was sequenced following a paired-end sequencing strategy with Illumina TruSeq Nano as well as the PacBio RS II platform. Both of them were provided by the sequencing service of Macrogen Inc. (South Korea). The Illumina sequencing libraries were prepared with the TruSeq Nano DNA kit with a read length of 101 nt, which produced a total of 3,949,108 paired-end reads with a %GC of 47.13%. The SMRTbell prep kit 3.0 was used to prepare the libraries of 10 to 40 kb long for PacBio II sequencing with a real-time observation of SMRT^R^-Cell, which generated 103,566 reads and an N_50_ value of 17,736. For both platforms, the trimming and quality control was performed with Fastp with default parameters (5). The reads of both platforms were assembled in a hybrid mode with default parameters using SPAdes v3.15.3 (6). The polypeptide sequence of orf2284 of *F. oxysporum* f. sp. *niveum* Fon020 (SNU77731) (3) was used as a query to search for a contig with the mtDNA using the TBLASTN algorithm (7). The contig that displayed higher nucleotide identity was selected. The circular architecture was verified by identifying a 55-nt overlapping sequence at both ends of the contig. The complete genome assembly is 45,675 bp with a %GC of 32.1 and harbors 18 protein-coding genes, two of them of unknown function, two rRNA-encoding genes, and 26 tRNA-encoding genes, all of them encoded in the sense strand (Table 1). The open reading frames were identified using the ORFfinder web version (https://www.ncbi.nlm.nih.gov/orffinder/) with the yeast mitochondrial genetic code (8) and confirmed by alignments with the mtDNA from *F. oxysporum* f. sp. *cubense* N2 (LT906350) and Fon020 (LT906340) whose annotation is reported (4). The position and identity of tRNA-encoding genes were determined using tRNAscan-SE (9) and by alignments with the mitogenomes mentioned above. Two Group I introns interrupting *nad5* and *rnl* were identified. The LAGLIDADG endonuclease (*heg1*) and the ribosomal protein S3 (*rps3*) are open reading frames encoded into the introns of *nad5* and *rnl*, respectively. The intron of *nad5* and the *heg1* encoded within are highly conserved in *F. oxysporum* mitogenomes (3). A diagram of the mtDNA of M5 is presented in Fig. 1.

**TABLE 1:** Genes identified in the mitochondrial genome of *Foc*M5^*a*^

Gene	Coordinates
*nad2*	1–1,665
*nad3*	1,666–2,079
*atp9*	2,936–3,160
*cox2*	4,238–4,987
*trnR* (acg)	5,120–5,190
*nad4L*	5,840–6,109
*nad5*	6,109–6,825, 7,836–9,107
intron IB	6,826–7,835
*heg1*	6,859–7,740
*trnR* (tct)	9,506–9,574
*cob*	10,949–12,121
*trnC* (gca)	12,324–12,396
*cox1*	13,764–15,356
*trnR* (tct)	15,624–15,694
*rf151*	16,443–16,898
*nad1*	17,323–18,432
*nad4*	18,720–20,177
*atp8*	20,313–20,459
*atp6*	20,952–21,752
*rns* (12S rRNA)	22,365–24,028
*trnY* (gta)	24,065–24,148
*trnD* (gtc)	24,149–24,206
*trnS* (gct)	24,452–24,533
*trnN* (gtt)	24,934–25,004
*cox3*	25,050–25,859
*nad6*	26,758–27,429
*trnV* (tac)	27,732–27,803
*trnI* (gat)	27,929–28,000
*trnS* (tga)	28,002–28,089
*trnW* (tca)	28,090–28,161
*trnP* (tgg)	28,171–28,243
rnl (16S rRNA)	28,591–31,297, 33,521–34,152
intron IA	31,298–33,520
*rps3*	31,727–33,154
*trnT* (tgt)	34,418–34,488
*trnE* (ttc)	34,549–34,620
*trnM* (cat)	34,622–34,692
*trnM* (cat)	34,693–34,762
*trnG* (tcc)	34,771–34,841
*trnL* (taa)	35,194–35,275
*rf2284*	35,975–42,829
*trnA* (tgc)	43,097–43,168
*trnF* (gaa)	43,174–43,246
*trnK* (ttt)	43,642–43,714
*trnL* (tag)	44,188–44,270
*trnQ* (ttg)	44,781–44,852
*trnH* (gtg)	45,058–45,130
*trnM* (cat)	45,537–45,609

^*a*^ The anticodon sequence is specified in the parentheses for every tRNA gene. Note that one intron interrupts both *nad5* and *rnl*, and one coding region is located within each intron.

## Data Availability

The raw data of Illumina and PacBio sequencing are deposited in SRA with the accession numbers SRX24377937 and SRX24377938, respectively. The nucleotide sequence of the mitochondrial genome of *Foc*M5 has been deposited in the GeneBanK under the accession number PP531459.
